# Alanyl‐Glutamine Attenuates Soybean Meal‐Induced Intestinal Dysfunction and Growth Retardation in Largemouth Bass (*Micropterus salmoides*)

**DOI:** 10.1155/anu/7842137

**Published:** 2025-12-19

**Authors:** Xinpeng Wang, Rongyan Yue, Jun Wen, Haiqing Wu, Xinghua Zhou, Yongjun Chen, Li Luo, Shimei Lin, Qinghui Ai, Yuanfa He

**Affiliations:** ^1^ College of Fisheries, Southwest University, Chongqing, 400715, China, southwest.edu; ^2^ Key Laboratory of Aquaculture Nutrition and Feed (Ministry of Agriculture and Rural Affairs), Key Laboratory of Mariculture (Ministry of Education), Ocean University of China, Qingdao, 266000, Shandong, China, ouc.edu.cn

**Keywords:** glutamine, intestinal microbiota, *Micropterus salmoides*, soybean meal-induced enteritis, transcriptomics

## Abstract

This study investigated the mitigating effects of alanyl‐glutamine (AG) on soybean‐meal‐induced enteritis (SBMIE) in largemouth bass (*Micropterus salmoides*). Three experimental diets were prepared: a fishmeal (FM) diet as a positive control, a 50% soybean meal (SBM) replacement FM protein (SBM50) diet as a negative control, and the SBM50 diet supplemented with 1% AG (SBM50 + 1% AG). Fish (initial weight: 10.20 ± 0.20 g) were distributed into three groups in triplicate (25 fish per tank) and fed for 8 weeks. Results demonstrated that the SBM50 + 1% AG group exhibited markedly higher final body weight, weight gain rate, and specific growth rate compared to the SBM50 (*p* < 0.05). The SBM50 + 1% AG group markedly elevated serum levels of free glycine, lysine, and total essential amino acids compared to the FM group (*p* < 0.05). In addition, the SBM50 + 1% AG group markedly increased the intestinal plica height (PH) and goblet cell numbers compared to the SBM50 group (*p* < 0.05). Pathological alterations, including villous atrophy, nuclear pyknosis, mitochondrial matrix dissolution, and inner membrane disruption, were shown in the SBM50 group, all of which were ameliorated by AG supplementation. In addition, the addition of AG significantly reduced Caspase3 activity compared to the FM group (*p* < 0.05). Microbiome analysis revealed dietary AG significantly increased *α*‐diversity and the proliferation of potentially beneficial taxa (Bacteroidota, *Bacteroides*, and *Prevotella*) (*p* < 0.05). Transcriptomics showed dietary AG upregulated intestinal barrier‐related pathways (including focal adhesion, cell adhesion molecules, and adherens junction), along with tight junction gene expression (*zo-1*, *claudin-3*, and *filamin-B*). In conclusion, high dietary SBM inclusion impairs growth performance and induces intestinal inflammation in largemouth bass. Dietary AG effectively mitigates SBMIE by remodeling the intestinal microbiota, enhancing intestinal barrier integrity, and modulating immune responses.

## 1. Introduction

Soybean meal (SBM) is a prominent plant‐derived protein source in aquatic feeds and is widely used to replace fishmeal (FM) owing to its well‐balanced amino acid composition and reliable availability [1]. Growth performance and intestinal health of fish will be maintained when fishmeal is replaced by SBM at moderate inclusion levels without adverse effects [2, 3]. However, high replacement levels have been documented to significantly suppress growth and damage intestinal health [4]. The replacement of 40% FM with SBM reduced the intestinal barrier in rainbow trout (*Oncorhynchus mykiss*), inducing intestinal inflammation [5]. Intestinal health was impaired in hybrid grouper (*Epinephelus fuscoguttatus* ♀ × *E. lanceolatus* ♂) following a 50% substitution of FM with SBM, primarily through compromised barrier function and shifts in microbial community abundance [6]. Studies also demonstrated that at a 45% FM substitution level with SBM, large yellow croaker (*Larimichthys crocea*) [7] exhibited a reduced intestinal barrier and upregulated *il-1*β and *tnf-α* expression.

Due to the intestinal inflammation induced by high‐dose SBM, it is necessary to identify dietary additives through nutritional strategies to alleviate SBM‐induced enteritis (SBMIE). Glutamine (Gln) serves as a crucial energy source and participates in various physiological reactions within the organism, including protein biosynthesis [8]. Owing to its roles in immunoregulation, Gln has been extensively employed in treating inflammatory bowel disease in mammals [9, 10]. Research in fish has demonstrated that Gln can ameliorate intestinal damage in zebrafish (*Danio rerio*) [11] and turbot (*Scophthalmus maximus*) [12]. Studies on hybrid grouper [13] demonstrated that Gln supplementation enhances intestinal immunity, modulates the abundance of intestinal microbiota, and mitigates intestinal inflammation induced by glycinin. However, research on the potential of Gln to alleviate SBMIE remains limited in fish, particularly in carnivorous species, and its underlying mechanisms remain to be further elucidated.

Largemouth bass (*Micropterus salmoides*) represents a commercially significant freshwater species widely cultured in China, renowned for its fast growth, nutritional quality, and desirable flesh taste [14]. This study evaluated the ameliorative effects of Gln against SBMIE in largemouth bass by establishing an experimental SBMIE model (replacing 50% of FM with SBM) through our previous research [15] and elucidating the underlying mechanisms of Gln’s protective effects. Furthermore, due to the thermal sensitivity of Gln, alanyl‐glutamine (AG) was used instead of Gln in this study, and AG exhibits similar physiological functions to Gln but possesses superior thermal stability [16]. The findings offer valuable insights for the application of SBM in aquafeeds and offer references for the nutritional regulation of intestinal health in cultured fish species.

## 2. Materials and Methods

### 2.1. Experimental Diets

Three diets were prepared to be isonitrogenous and isolipidic: a positive control (FM), a negative control (SBM50), and an experimental diet (SBM50 + 1% AG). The inclusion level of AG was determined based on previous research [17, 18]. All ingredients were finely ground through a 60‐mesh sieve. Minor components were blended by gradual dilution and mixing before incorporation into bulk ingredients. After homogenization with fish and soybean oils, the mixture was pelleted into 2.0 mm diameter diets using a twin‐screw extruder (SG‐YPYS‐76, Xiamen Xinyuanfa Machinery Equipment Factory, China). The prepared diets were air‐dried and stored at −20°C until use. The proximate composition was analyzed following AOAC [19]. The feed formulations are presented in Table 1.

**Table 1:** Ingredients and nutrition level of experimental diets (g/kg)

Ingredients	Groups			
	FM	SBM50	SBM50+1% AG	
Fish meal	420.00	210.00		210.00
Soybean meal^a^	0.00	282.80		282.80
Chicken meal	120.00	120.00		120.00
Plasma protein meal	50.00	50.00		50.00
Porcine meat meal	50.00	50.00		50.00
Peanut bran	70.00	70.00		70.00
Wheat flour	20.00	20.00		20.00
Fish oil	20.00	20.00		20.00
Soybean oil	15.00	25.00		25.00
Bentonite	80.50	39.20		39.20
Ca(H_2_PO_4_)_2_	15.00	15.00		15.00
Choline chloride	5.00	5.00		5.00
Vitamin C (35%)	1.00	1.00		1.00
Vitamin and mineral premix^b^	20.00	20.00		20.00
Ethoxyquin	0.50	0.50		0.50
Alanyl‐glutamine^c^	0.00	0.00		10.00
Alanine^c^	20.00	20.00		10.00
Non‐essential amino acids	10.00	0.00		0.00
Lysine^c^	0.50	8.40		8.40
Methionine^c^	1.90	3.90		3.90
Microcrystalline cellulose	80.60	39.20		39.20
Total	1000.00	1000.00		1000.00
Nutritional level^d^				
Crude protein	498.70	498.60		498.60
Crude lipid	115.10	112.10		112.10
Lysine level	31.40	37.80		38.10
Methionine level	10.80	11.10		11.00

^a^Crudeprotein, 46.31%; Crude lipid, 1.08%; Crude ash, 5.91%; Crude fiber, 5.12%.

^b^Thepremix were purchased from Chongqing CITICO Biotech Co., Ltd. (Chongqing,China).

^c^Purchased from Shanghai Sanjie Biotech Co., Ltd. (Shanghai, China).

^d^Allnutrition levels were actually measured values.

### 2.2. Experimental Procedures

Fish were purchased from a commercial farm (Chongqing Sanxia Ecological Fisheries Co., Ltd.; China) and acclimatized for 2 weeks while being fed a commercial diet (Foshan Jieda Feed Co., Ltd.; China). Following acclimatization, healthy largemouth bass (initial weight: 10.20 ± 0.20 g) were distributed into three groups in triplicate (25 fish per tank). The feeding trial was conducted from May 12, 2024, to July 6, 2024 (an 8‐week period). Fish were fed to apparent satiation twice daily at 09:00 and 17:00. The feces were removed by siphoning at midday, and 50% of the tank water was replenished daily with pre‐aerated water. The water quality parameters were maintained as follows: dissolved oxygen > 6.0 mg/L, total ammonia nitrogen < 0.2 mg/L, temperature was 28°C–30°C, and pH was 7.4–7.8.

### 2.3. Sample Collection

After the 8‐week feeding, all fish were fasted for 24 h and subsequently euthanized with buffered tricaine methanesulfonate (MS‐222; Sigma, USA) at 300 mg/L [20]. Each fish was individually weighed to evaluate growth performance. Subsequently, nine fish per tank were randomly sampled for body length measurement and blood collection. Intestine weight, intestine length, viscera weight, and hepatic weight were recorded for subsequent morphologic parameter calculation. Serum was obtained without anticoagulants by clotting samples at 4°C for 24 h, followed by centrifugation at 4°C, 3000 rpm for 10 min. The supernatant was aliquoted and stored at −80°C for further analysis. For histological examination, ~1 cm segments of distal intestine from 4 fish per tank were excised and immediately fixed in either 4% neutral‐buffered paraformaldehyde (for hematoxylin and eosin and periodic acid‐Schiff [PAS] staining) or 2.5% glutaraldehyde (for transmission electron microscopy [TEM]). Additional distal intestine of four fish per tank for enzymatic activity assays. Four content‐free distal intestines of fish from each tank were taken for microbiome analyses, with two intestines pooled to form one sample. Four distal intestines per tank were taken for transcriptome and qPCR analyses, in which two intestines were pooled to form one sample.

### 2.4. Quantification of Serum Free Amino Acid Contents.

Serum (50 *μ*L) was mixed with an equal volume of 5% (w/v) sulfosalicylic acid for deproteinization. After ice‐cold incubation, the mixture was centrifuged at 12,000 rpm for 15 min at 4°C. The resulting supernatant was pH‐adjusted to 2.2 and reconstituted to 50 *μ*L using 0.2 M sodium citrate buffer (pH 2.2) before analysis.

### 2.5. Intestinal Histology and TEM Analysis

To preserve ultrastructural integrity, distal intestine samples were immersed in 4% paraformaldehyde. Fixed tissues were dehydrated through a graded ethanol series, cleared in xylene to remove residual ethanol until fully transparent, and embedded in paraffin. Serial sections (4 µm) were prepared using a rotary microtome for hematoxylin and eosin (H&E) and PAS staining. Ten measurements each of intestinal structure used for plica height (PH), plica weight, and muscular layer thickness (MLT) were obtained by ImageJ (NIH, USA). When measuring the above indicators, the experimenter did not know the group to which each section belonged to ensure the objectivity of the measurement results.

For TEM, three additional distal intestine tissues per tank were fixed in 2.5% glutaraldehyde. After rinsing with phosphate buffer, the tissues were post‐fixed in 1% osmium tetroxide, dehydrated through an ethanol series, and embedded in epoxy resin. Following polymerization at 60°C for 48 h, ultrathin sections were prepared using an ultramicrotome, stained with uranyl acetate and lead citrate, and examined via TEM.

### 2.6. Analysis of Intestinal Immunity Parameters

Distal intestine tissues were homogenized in physiological saline (1:9, w/v) with a bead‐based homogenizer at 4°C. The homogenate was centrifuged at 12,000 rpm for 15 min (4°C), and the supernatant was collected for subsequent assays. Concentrations of intestinal immune mediators, including complement component 3 (C3; #YJ003460), complement component 4 (C4; #YJ003461), caspase‐3 (#YJ592313), caspase‐9 (#YJ593782), and antimicrobial peptides (AMPs; #YJ203750), were quantified following the manufacturer’s instructions (Shanghai Enzyme‐linked Biotechnology Co., Ltd.; China).

### 2.7. Intestinal Microbiome Analysis

Genomic DNA was obtained from distal intestinal content using a kit (Magen, Guangzhou, China). The V3–V4 region of the bacterial 16S rRNA gene was PCR‐amplified with universal primers 341F (5^′^‐CCTACGGGNGGCWGCAG‐3^′^) and 806R (5^′^‐GGACTACHVGGGTWTCTAAT‐3^′^). Amplified products were examined on 2% agarose gels. Libraries were prepared with the Illumina DNA Prep Kit (Illumina, CA, USA) and subjected to paired‐end sequencing on an Illumina platform. (Gene Denovo Biotechnology Co., Ltd., Guangzhou, China).

Raw sequencing data were processed with UPARSE (v9.2.64) to generate operational taxonomic units (OTUs) at 97% sequence similarity. Taxonomy was assigned using the SILRA database (v138.1). Alpha diversity was computed in QIIME (v1.9.1).

### 2.8. Transcriptome Analysis

Total RNA was isolated from intestinal tissues with TRIzol Reagent (Invitrogen, USA). RNA integrity, purity, and concentration were assessed using a NanoDrop 2000 spectrophotometer (Thermo Fisher Scientific, USA). cDNA libraries were prepared from qualified RNA and sequenced on the Illumina NovaSeq 6000 platform (Illumina Inc., USA; Gene Denovo Biotechnology Co., Ltd, Guangzhou, China).

Raw reads underwent control with fastp (v0.23.2), including low‐quality bases and adapter sequences. Ribosomal RNA (rRNA) was subsequently filtered using Bowtie2 (v2.2.8). High‐quality remaining reads were aligned to the largemouth bass reference genome (ASM1485139v1) using HISAT2 (v2.2.4). Transcript assembly and quantification were performed with RSEM (v1.3.3). Significantly differentially expressed genes (DEGs) are defined as those meeting |log_2_(fold change)| > 1 and false discovery rate (FDR) ≤ 0.05. DEGs were subjected to functional enrichment analysis based on Gene Ontology (GO) and the Kyoto Encyclopedia of Genes and Genomes (KEGG) pathway database with the clusterProfiler package (v4.2.2) in R.

### 2.9. Quantitative Real‐Time PCR Validation

Total RNA was extracted from intestinal tissue samples with TRIzol Reagent (Invitrogen, USA). RNA concentration and purity were determined using a NanoDrop 2000 spectrophotometer (Thermo Fisher Scientific, USA). The A260/280 values of RNA ranged between 1.8 and 2.0. cDNA was synthesized using the PrimeScript RT Reagent Kit (RR092A; Takara, Japan). All qPCR primers (Table 2) were designed by the primer blast in the National Center for Biotechnology Information (NCBI) and manufactured by Sangon Biotech (Shanghai, China). Quantitative real‐time PCR was carried out using TB Green Premix Ex Taq II (RR820A; Takara, Japan) on a CFX96 Touch Real‐Time PCR system (Bio‐Rad, USA). The *eef1α1* gene served as the internal control [21], and the relative expression was calculated by the 2^−*ΔΔ*CT^ method [22].

**Table 2:** Detailsof primers for qPCR.

Gene	Primer sequence (5’‐3’)	Product size (bp)	GenBank
*zo-1*	F: CGGCTGCCCTGTTCCCAG R: AACCACTTCGACAGGGACCA	128	XM_038701018.1
*occludin*	F: GCCAGAACCTGTACCAGACC R: GAAAGCTGCAACACCCAAGG	161	XM_038715418.1
*p50*	F: TCAGAAAACAGGAAACGGAAGT R: GTGACATTTCCAAAGCCTCCG	146	XM_038702563.1
*desmin*	F: ACATGCGCGCAGAGATAGAA R: CTTGGTTGTGCAAGGTCGTG	156	XM_038695924.1
*filamin*	F: TTCCAAGTCACGGCAACAGA R: CTGATGGCATTCGGACCTCA	133	XM_038723071.1
*eef1α1*	F: GTTGCTGCTGGTGTTGGTGAG R: GAAACGCTTCTGGCTGTAAGG	156	XM_038724778.1

*Note:* p50: nuclear factor kappa B subunit 1, eef1*α*1: eukaryotic translation elongation factor1‐alpha 1.

Abbreviation: zo‐1, zonula occludens‐1.

### 2.10. Calculations and Statistical Analysis

The calculation formula for the target parameters is as follows:

Weight gain rateWGR,%=100×final weight− initial weight/initial weight.

Specific growth rateSGR,%/d=100×lnfinal weight−lninitial weight/56d.

Feed efficiency ratioFER=final weight− initial weight/feed intake.

Viscera somatic indexVSI,%=100×visceral weight/fish weight.

Hepatopancreas somatic indexHSI,%=100×hepatic weight/fish weight.

Intestine somatic indexISI,%=100×intestinal weight/fish weight.

Relative gut lengthRGL,%=100×intestinal length/body length.

Mesenteric fat indexMFI,%=100×mesenteric fat weight/initial weight.

Condition factorCF=100×final weight/body length3.

Data were analyzed via Excel 2019 and SPSS 27.0. Graphs were generated using GraphPad Prism 8.0.2. Statistical analyses were performed using one‐way ANOVA and Duncan’s multiple comparison test. Results are expressed as mean ± SEM, and differences were considered statistically significant at *p* < 0.05.

## 3. Results

### 3.1. Growth Performance and Physical Indices

The SBM50 + 1% AG group showed significantly improved FBW, WGR, and SGR compared to the SBM50 group (*p* < 0.05, Table 3). In contrast, FE did not differ markedly among the treatments (*p* > 0.05). Relative to the FM group, the SBM50 group markedly reduced VSI, RGL, and CF (*p* < 0.05). No significant differences were shown in HSI or ISI across dietary treatments (*p* > 0.05).

**Table 3:** Effects of dietary glutamine on growth performance and physical indices of largemouth bass fed with high soybean meal diets.

Items	Groups		
	FM	SBM50	SBM50+1% AG
FBW (g)	51.25±1.13^ab^	48.92±0.45^a^	53.91±0.85^b^
WGR (%)	393.16±10.60^ab^	369.53±4.12^a^	418.25±8.69^b^
SGR (%)	2.85±0.04^ab^	2.76±0.02^a^	2.94±0.03^b^
FE (%)	1.12±0.02	1.12±0.02	1.10±0.01
VSI (%)	6.53±0.13^b^	6.07±0.11^a^	6.13±0.14^a^
HSI (%)	0.64±0.06	0.83±0.12	0.73±0.08
ISI (%)	0.86±0.04	0.60±0.01	0.91±0.19
RGL (%)	76.45±1.98^b^	71.23±0.58^a^	72.10±0.98^ab^
CF (g/cm^3^)	2.06±0.03^b^	1.97±0.02^a^	1.99±0.04^ab^

*Note:* Values (means ± SEM, *n* = 3) in the same row with different letter superscripts differ markedly (*p* < 0.05).

Abbreviations: CF, condition factor; FBW, final body weight; FE, feed efficiency; HSI, hepatopancreas somatic index; ISI, intestine somatic index; MFI, mesenteric fat index; RGL, relative gut length; SGR, specific growth rate; VSI, viscera somatic index; WGR, weight gain rate.

### 3.2. Serum Free Amino Acid Content

The SBM50 + 1% AG group exhibited significantly lower methionine, lysine, and histidine than the SBM50 group. (*p* < 0.05, Table 4). The levels of valine, isoleucine, leucine, threonine, and arginine showed no significant difference among groups (*p* > 0.05). The glycine was markedly higher in the SBM50 + 1% AG group compared to the SBM50 group among the non‐essential amino acids (NEAA) (*p* < 0.05). The contents of serine, glutamic acid, tyrosine, glutamine, and *γ*‐aminobutyric showed no marked difference among the three groups (*p* > 0.05). The total contents of NEAA (*Σ*NEAA) and total free amino acids (*Σ*TFAA) did not differ significantly across treatments (*p* > 0.05).

**Table 4:** Effects of dietary glutamine on serum free amino acid contents of largemouth bass fed with high soybean mealdiets (mg/mL).

Amino acids	Groups		
	FM	SBM50	SBM50+1% AG
Essential amino acids			
Valine	0.036 ± 0.001	0.040 ± 0.005	0.043 ± 0.002
Methionine	0.017 ± 0.001^a^	0.017 ± 0.001^a^	0.021 ± 0.001^b^
Isoleucine	0.021 ± 0.000	0.023 ± 0.003	0.026 ± 0.001
Leucine	0.038 ± 0.001	0.042 ± 0.006	0.049 ± 0.001
Threonine	0.024 ± 0.002	0.030 ± 0.003	0.030 ± 0.003
Phenylalanine	0.022 ± 0.000^b^	0.020 ± 0.001^a^	0.020 ± 0.001^ab^
Lysine	0.013 ± 0.001^a^	0.026 ± 0.002^b^	0.039 ± 0.002^c^
Histidine	0.022 ± 0.000^ab^	0.020 ± 0.002^a^	0.024 ± 0.000^b^
Arginine	0.013 ± 0.000	0.013 ± 0.001	0.013 ± 0.001
*Σ*EAA	0.210 ± 0.004^a^	0.230 ± 0.022^ab^	0.260 ± 0.010^b^
Non‐essential amino acids			
Aspartic acid	0.019 ± 0.000^a^	0.022 ± 0.003^ab^	0.026 ± 0.001^b^
Serine	0.020 ± 0.002	0.025 ± 0.002	0.024 ± 0.001
Glutamic acid	0.022 ± 0.000	0.023 ± 0.001	0.022 ± 0.001
Glycine	0.078 ± 0.002^c^	0.061 ± 0.004^a^	0.069 ± 0.001^b^
Alanine	0.076 ± 0.002^a^	0.084 ± 0.008^ab^	0.094 ± 0.003^b^
Cystine	0.003 ± 0.000^b^	0.001 ± 0.000^a^	0.001 ± 0.000^a^
Tyrosine	0.025 ± 0.001	0.024 ± 0.003	0.028 ± 0.001
Proline	0.009 ± 0.001^a^	0.015 ± 0.002^b^	0.018 ± 0.001^b^
Tryptophan	0.005 ± 0.000^b^	0.004 ± 0.001^a^	0.003 ± 0.000^a^
Glutamine	0.018 ± 0.002	0.013 ± 0.002	0.016 ± 0.001
Taurine	0.144 ± 0.006^b^	0.103 ± 0.014^a^	0.109 ± 0.002^a^
*γ*‐Aminobutyric	0.012 ± 0.001	0.012 ± 0.001	0.010 ± 0.000
*Σ*NEAA	0.430 ± 0.008	0.380 ± 0.034	0.420 ± 0.004
*Σ*TFAA	0.635 ± 0.008	0.615 ± 0.055	0.682 ± 0.013

*Note:* Values (means ± SEM, *n* = 3) in the same row with different letter superscripts differ markedly (*p* < 0.05). *Σ*EAA: total essential amino acids; *Σ*NEAA: total non‐essential amino acids; *Σ*TFAA: total free amino acids.

### 3.3. Histological Examination and Intestinal Morphometry

Histological examination revealed that the SBM50 group exhibited more compromised intestinal morphology than the FM. In contrast to the SBM50, AG supplementation mitigated these morphological alterations (Figure 1A). The SBM50 group showed the lowest values in both MLT and PH, with the latter parameter being significantly reduced compared to both the FM and SBM50 + 1% AG (*p* < 0.05). No marked differences were detected in plica width (PW) across groups (*p* > 0.05; Figure 1B). Goblet cell counts were markedly reduced in the SBM50 relative to the FM, while the SBM50 + 1% AG markedly increased the goblet cell number more than the SBM50 (*p* < 0.05, Figure 1B).

Figure 1Effects of dietary glutamine on distal intestinal histology in largemouth bass fed high soybean meal diets. (A) H&E and PAS staining results of three groups, scale bars 400 µm (4 ×), scale bars 200 µm (10 ×). (B) Intestinal microstructural morphological measurement among groups (means ± SEM, *n* = 3). Columns with different letters differ markedly (*p* < 0.05). MLT: muscular layer thickness; PH: plica height; PW: plica width.

**Figure:**
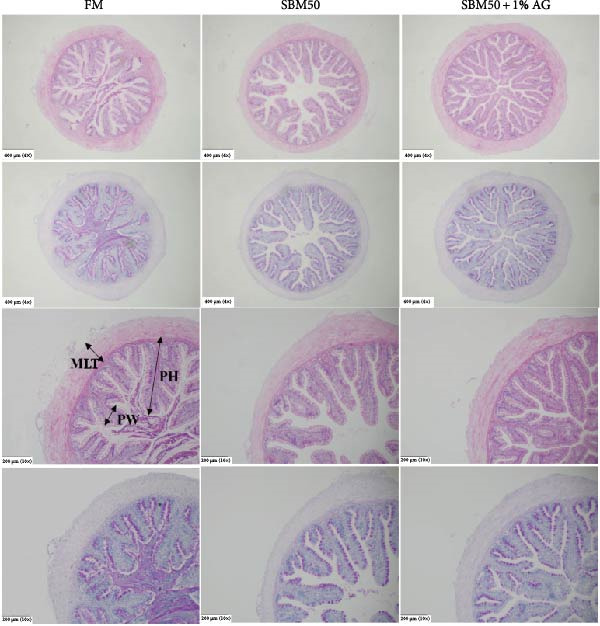
(A)

**Figure:**
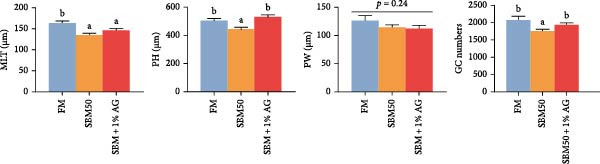
(B)

**Figure 2:**
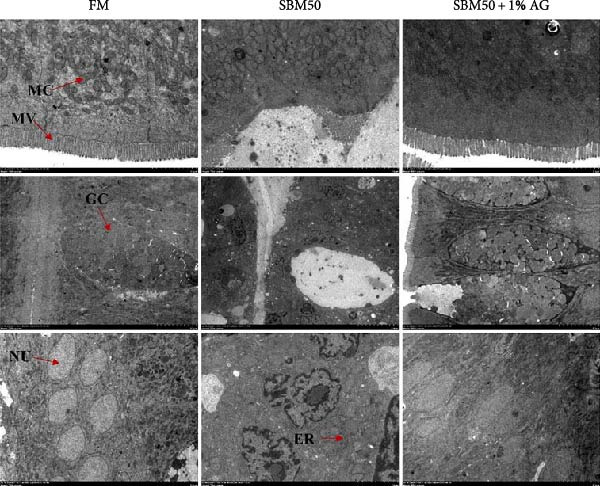
Effects of dietary glutamine on distal intestinal ultrastructure in largemouth bass fed high soybean meal diets. MV: microvilli; MC: mitochondria; GC: goblet cell; NU: nucleus; ER: endoplasmic reticulum.

TEM showed that the SBM50 group exhibited pathological alterations including intestinal microvilli sloughing, nuclear pyknosis, vacuolation of goblet cells resulting from dissolution of mucigen granules, dissolution of mitochondrial matrix and double membranes, and dilation and fragmentation of the endoplasmic reticulum (Figure 2). In contrast, both the SBM50 + 1% AG group and the FM group displayed relatively normal intestinal microvilli and cellular ultrastructure (Figure 2).

### 3.4. Intestinal Immunity

Caspase3 content was highest in the SBM50 + 1% AG group, significantly exceeding that in the other groups (*p* < 0.05; Figure 3). In contrast, caspase9, C3, C4, and AMPs levels showed no marked differences across treatments (*p* > 0.05, Figure 3).

Figure 3Effects of dietary glutamine on intestinal immunity in largemouth bass fed high soybean meal diets. Columns with different letters differ markedly (means ± SEM, *n* = 3). (A) Caspase3: cysteine aspartate protease 3; (B) Caspase9: cysteine aspartate protease 9. (C) C3: complement C3; (D) C4: complement C4; (E) AMPs: antimicrobial peptides.

**Figure:**
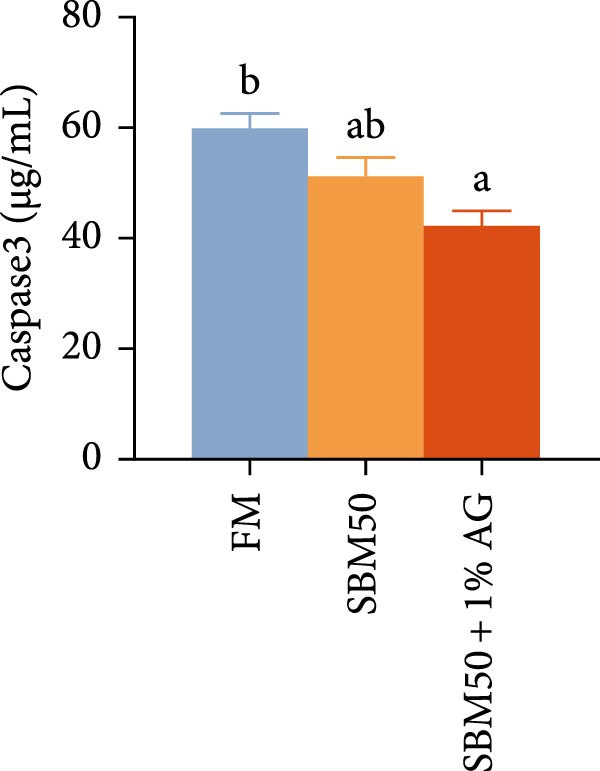
(A)

**Figure:**
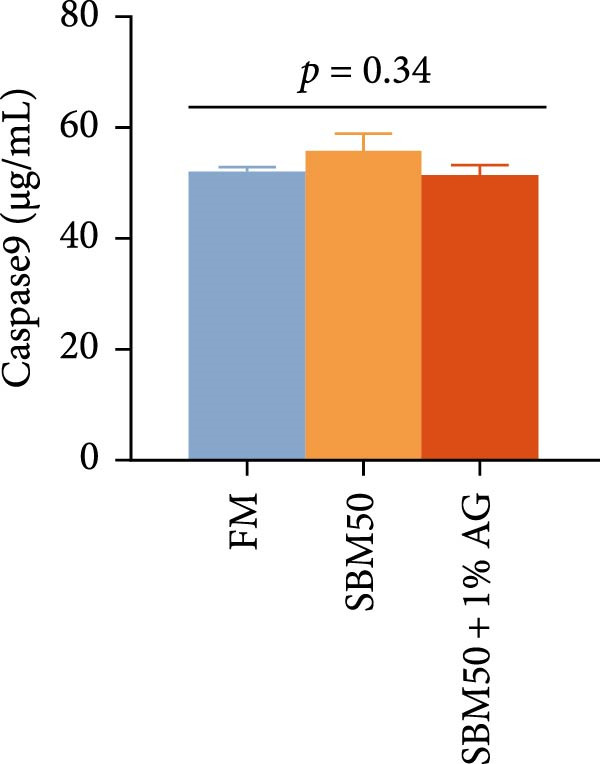
(B)

**Figure:**
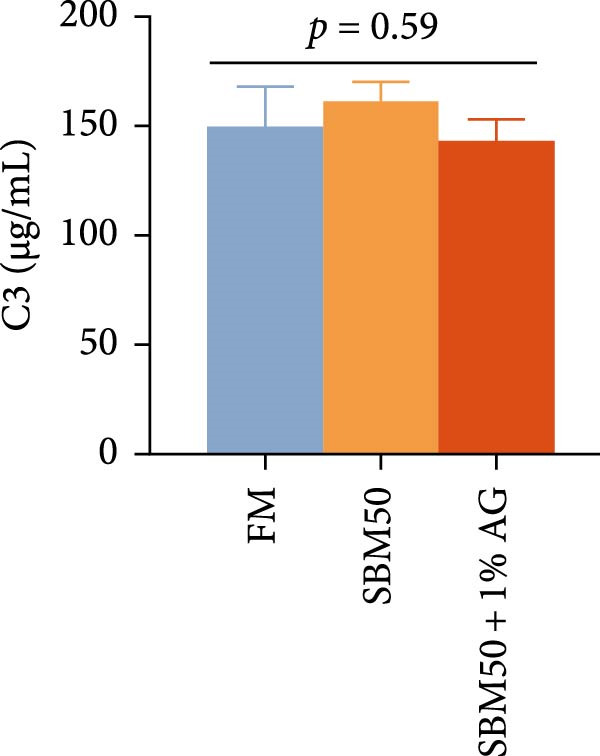
(C)

**Figure:**
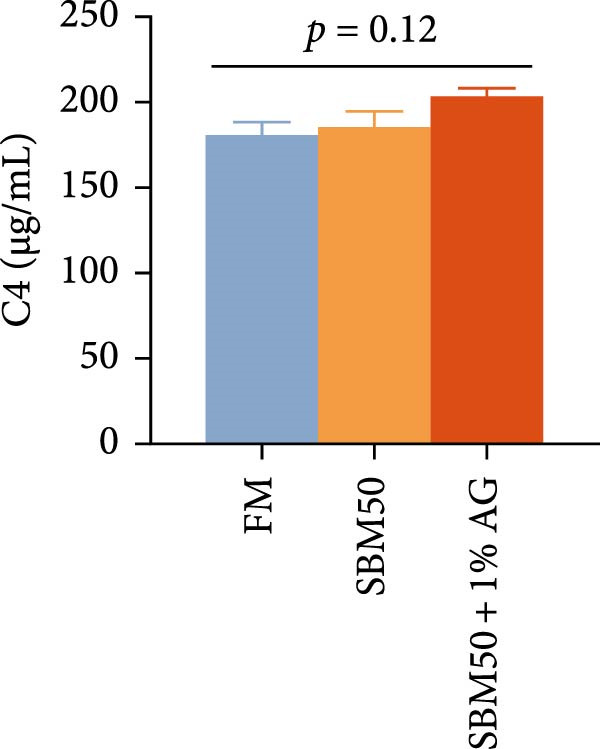
(D)

**Figure:**
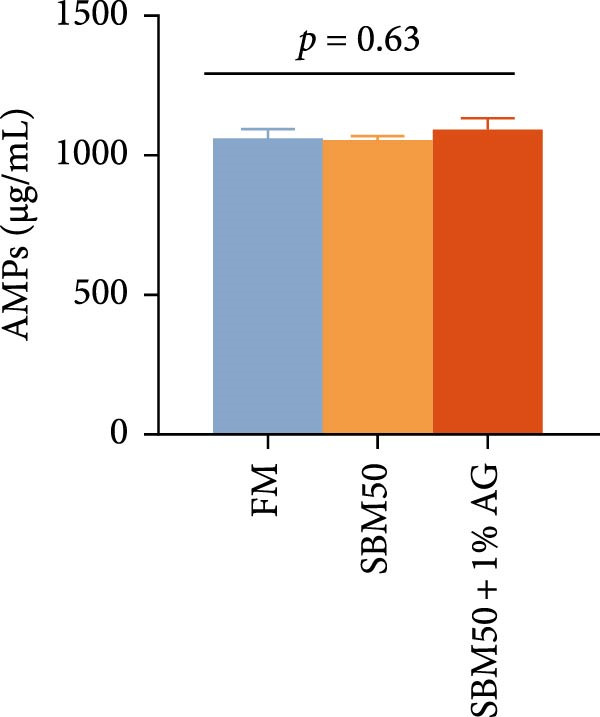
(E)

### 3.5. Intestinal Microflora

**Figure 5:**
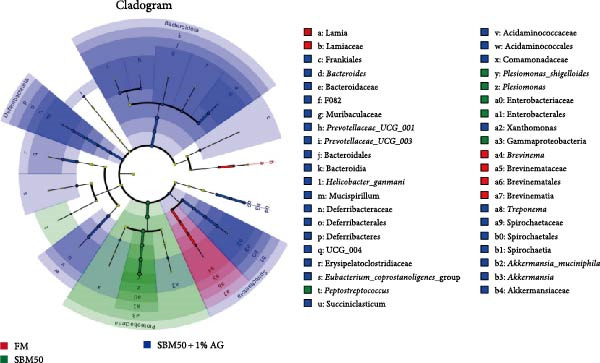
LEfSe analysis of glutamine’s effects on the intestinal microbiota in largemouth bass fed high soybean meal diets. This figure displays all significantly different microbial taxa (from domain to genus level) with a linear discriminant analysis (LDA) score > 3.

In the analysis of alpha diversity, the SBM50 + 1% AG group exhibited significantly elevated Pielou, Shannon, and Simpson indices compared to the FM and SBM50 groups (*p* < 0.05; Figure 4A), whereas the Chao1 index showed no notable differences across treatments (*p* > 0.05, Figure 4A). Beta diversity assessment via PCoA combined with species composition stacked plots demonstrated marked differences in microbial community composition (*p* < 0.05, Figure 4B,C,D). Phylum‐level analysis showed that the SBM50 markedly elevated the abundance of Proteobacteria compared to the FM and SBM50 + 1% AG groups (*p* < 0.05; Figure 4F), while Bacteroidota levels were notably higher in the SBM50 + 1% AG group than in the other treatments (*p* < 0.05; Figure 4F). Genus‐level analysis revealed notable decreased abundances of *Aeromonas* in the SBM50 + 1% AG relative to FM (*p* < 0.05, Figure 4G), while *Bacteroides*, *Treponema*, and *Prevotellaceae_UCG-001* showed markedly higher abundances in this treatment (*p* < 0.05, Figure 4G). Venn diagram analysis at the genus level indicated 272, 212, and 415 genera in the FM, SBM50, and SBM50 + 1% AG groups, respectively. (Figure 4E). LEfSe analysis identified Enterobacterales and Enterobacteriaceae as discriminant taxa for the SBM50 group, *Brevinema* for the FM group, and Bacteroidota with Deferribacterota for the SBM50 + 1% AG group (Figure 5), indicating that high SBM inclusion promoted potentially detrimental bacterial populations while AG supplementation enriched beneficial microbiota.

Figure 4Effects of glutamine on the intestinal microbiota in largemouth bass fed high soybean meal diets. (A) Species alpha diversity bar plot. (B) Phylum‐level taxonomic composition. (C) Genus‐level taxonomic composition. (D) Principal Coordinate Analysis plot. (E) Genus‐level Venn diagram. (F) Phylum‐level bacterial taxa. (G) Genus‐level bacterial taxa. Columns with different letters differ markedly (means ± SEM, *n* = 3).

**Figure:**
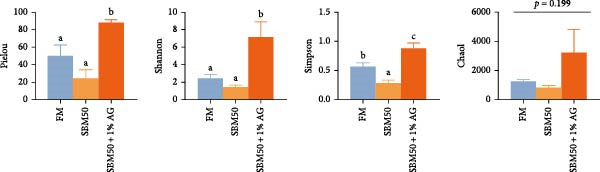
(A)

**Figure:**
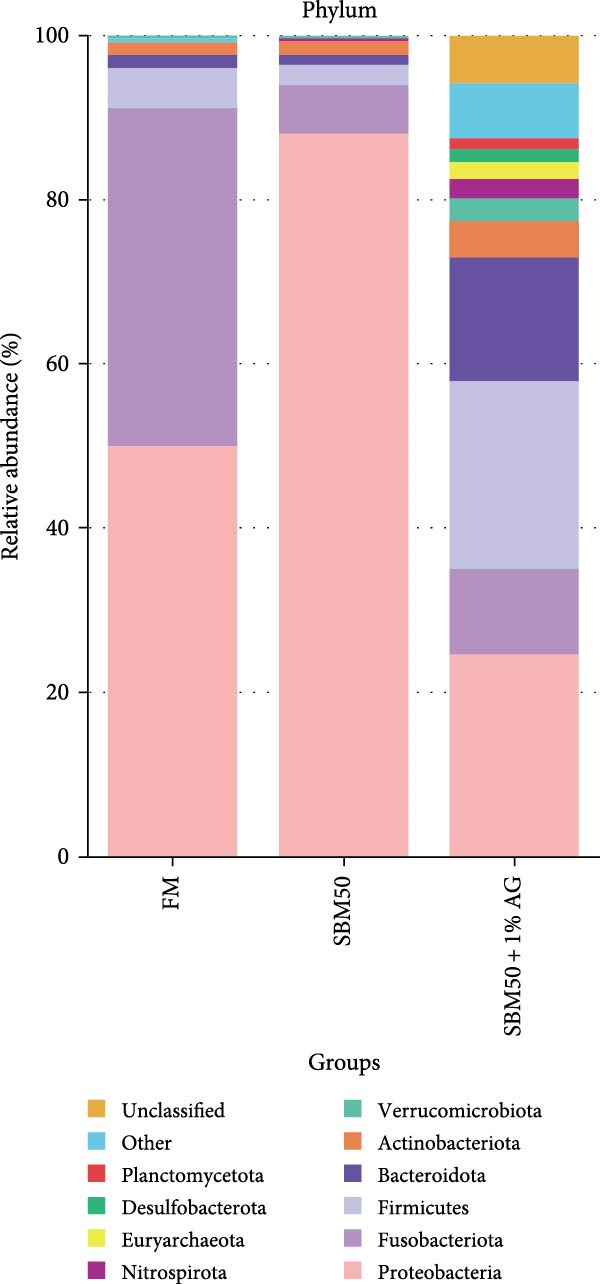
(B)

**Figure:**
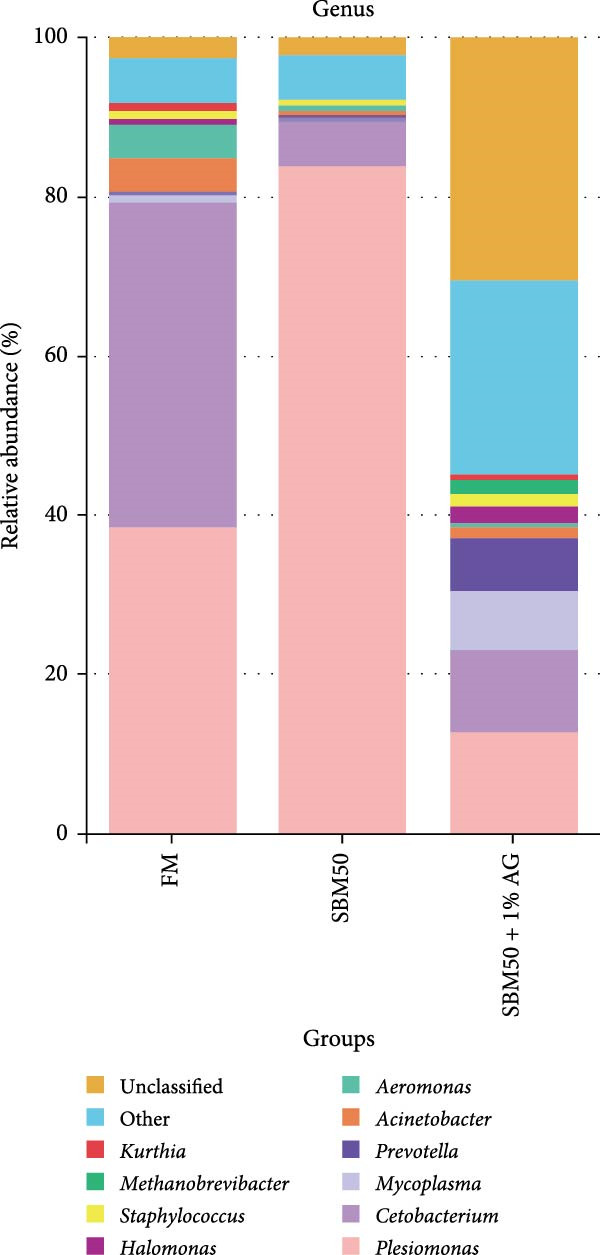
(C)

**Figure:**
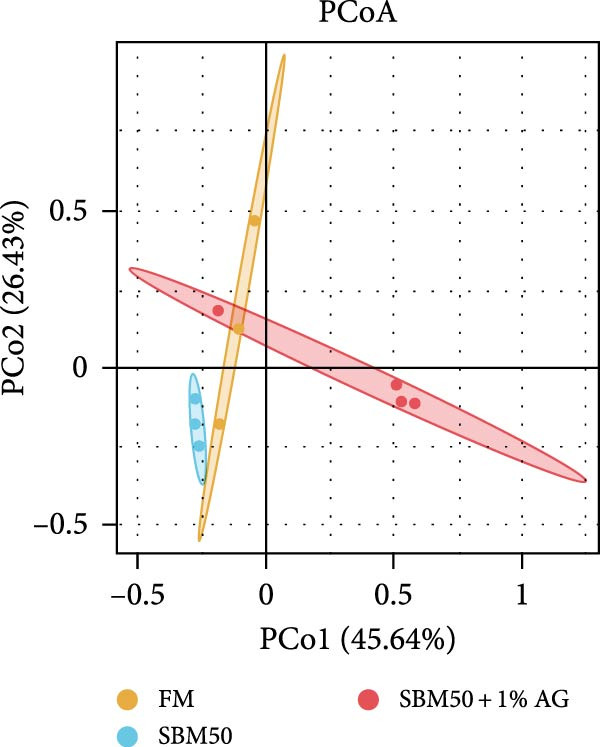
(D)

**Figure:**
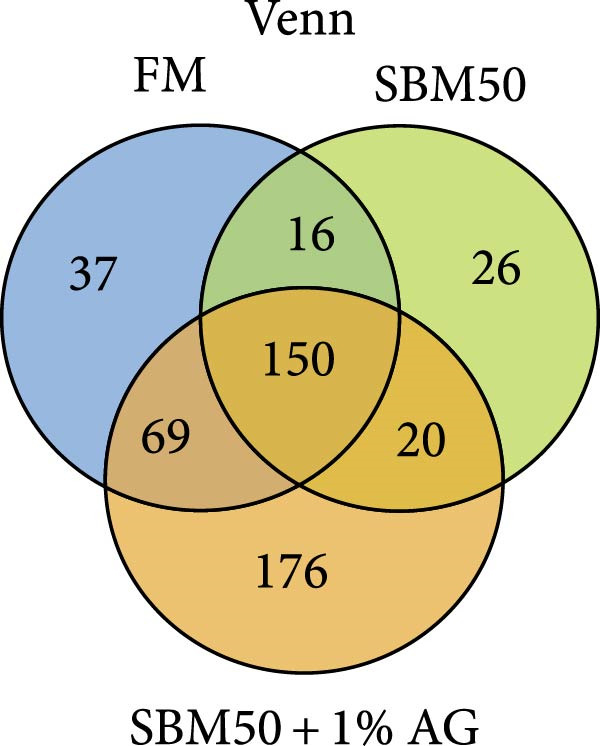
(E)

**Figure:**
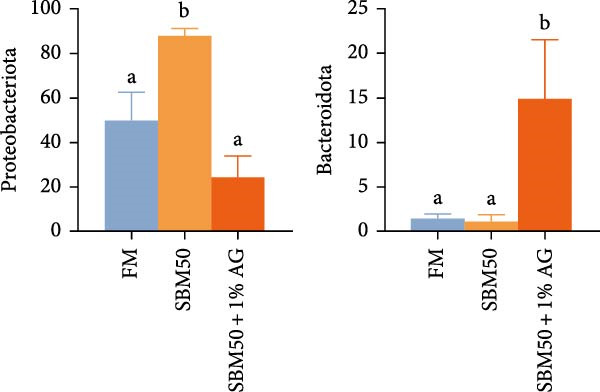
(F)

**Figure:**
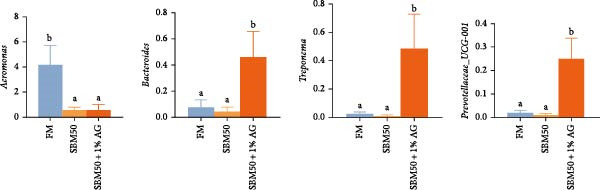
(G)

### 3.6. Transcriptome Analysis

PCA results indicated significant differences in gene expression profiles among groups (Figure 6A). Relative to the FM, the SBM50 exhibited 128 upregulated and 210 downregulated genes (FDR < 0.05, log_2_|FoldChange| > 1), while 2803 genes were upregulated and 1220 downregulated in the SBM50 + 1% AG versus the SBM50 (Figure 6B). Notable alterations occurred in intestinal barrier‐related genes: expression levels of *tubulin α*, *trypsin-2*, and *muc2* were notably lower in the SBM50 than in the FM (*p* < 0.05; Figure 6C,G), indicating that high SBM content induced intestinal damage in largemouth bass. In contrast, dietary AG supplementation significantly enhanced the genes’ expression (*zo-1*, *claudin-3*, *filamin-B*, *actinin*, *lamin-A*, and *integrin α*) relative to the SBM50 group (Figure 6F,G), demonstrating that AG supplementation notably enhanced molecular markers of intestinal barrier integrity. KEGG pathway analysis of FM versus SBM50 revealed that the top five enriched pathways were *Vibrio cholerae* infection, amino sugar and nucleotide sugar metabolism, arachidonic acid metabolism, starch and sucrose metabolism, and the hippo signaling pathway‐fly (Figure 6D). In contrast, comparison between SBM50 and SBM50 + 1% AG groups identified significant enrichment of three intestinal barrier‐related pathways (focal adhesion, cell adhesion molecules, and adherens junction) (Figure 6E).

Figure 6Effects of dietary glutamine on the intestinal transcriptome in largemouth bass fed high soybean meal diets. (A) Principal component analysis plot. (B) All significantly differentially expressed genes under the conditions FM vs SBM50 and SBM50 vs SBM + 1% AG. (C, F) Volcano plot of significantly DEGs. (D, E) KEGG pathway enrichment plots of significantly enriched pathways. (G) Bar plot of significantly DEGs related to intestinal barrier function. Significance is indicated by asterisks:  ^∗^(*p* < 0.05),  ^∗∗^(*p* < 0.01),  ^∗∗∗^(*p* < 0.001).

**Figure:**
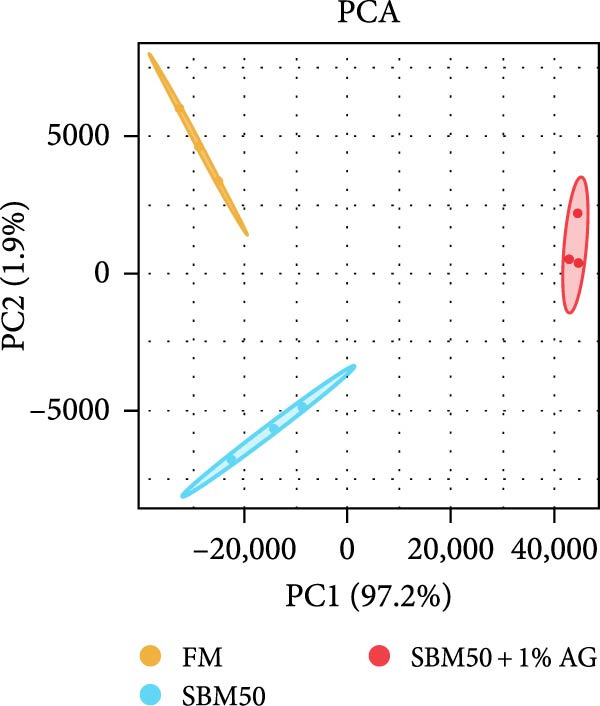
(A)

**Figure:**
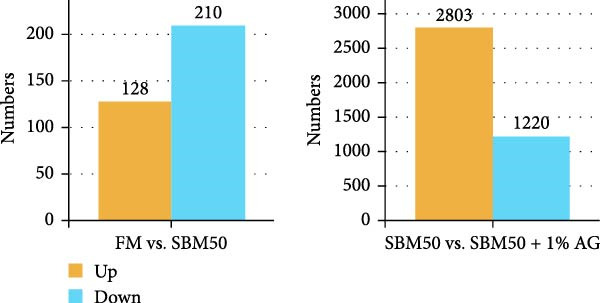
(B)

**Figure:**
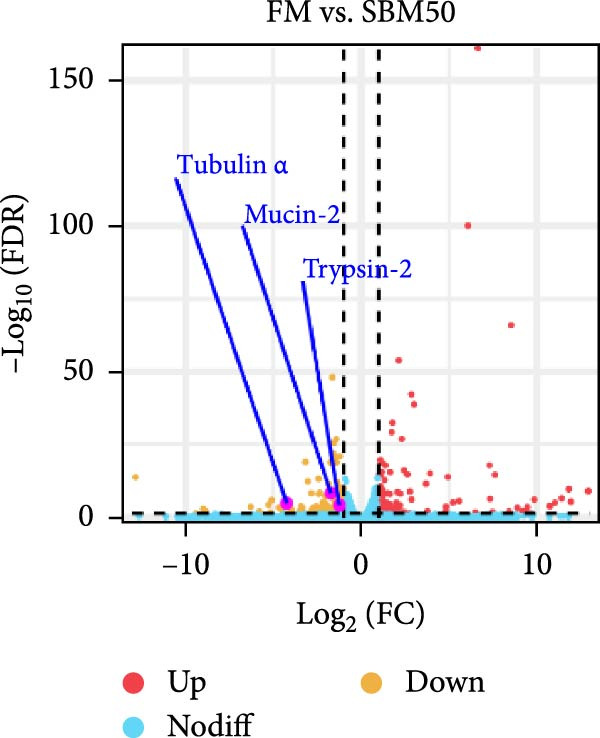
(C)

**Figure:**
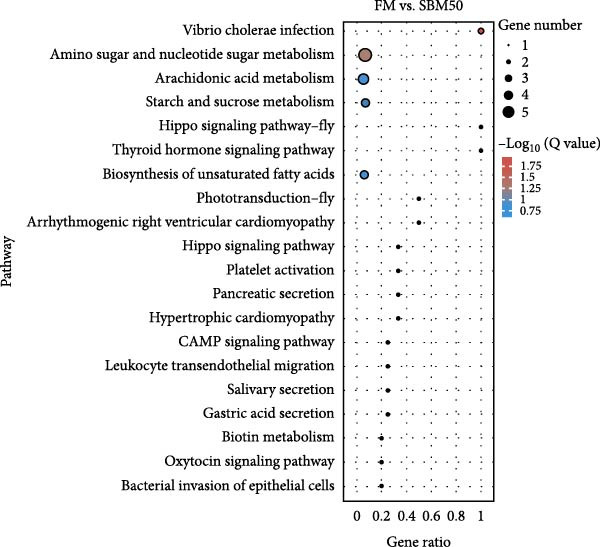
(D)

**Figure:**
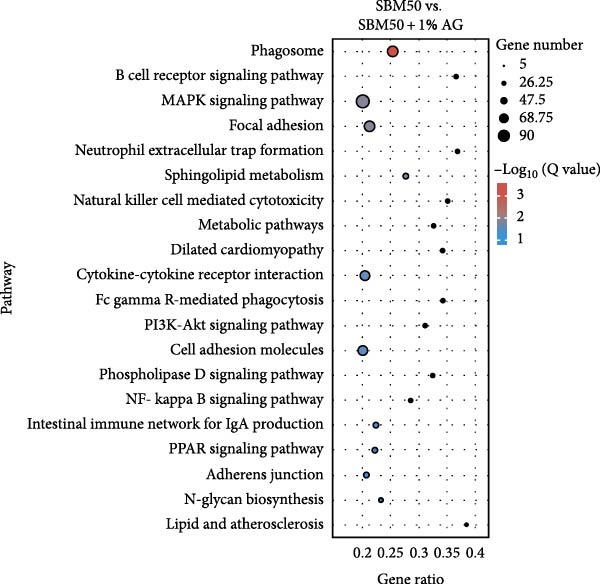
(E)

**Figure:**
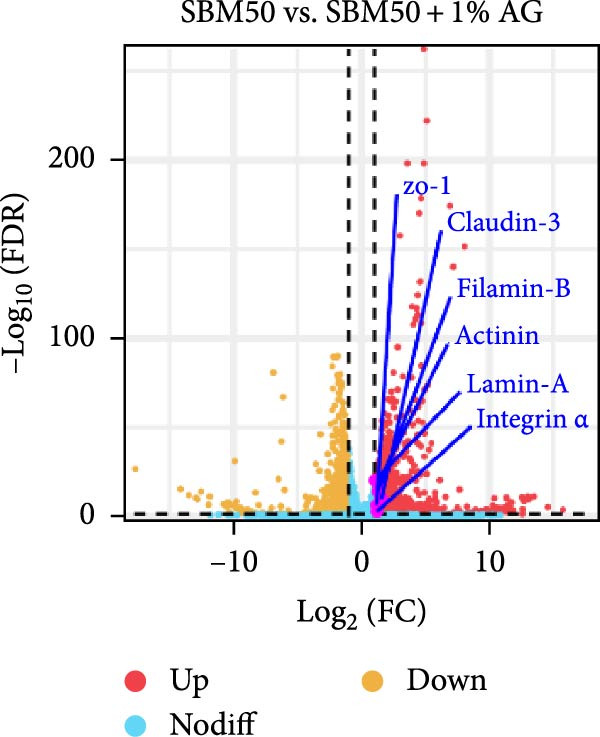
(F)

**Figure:**
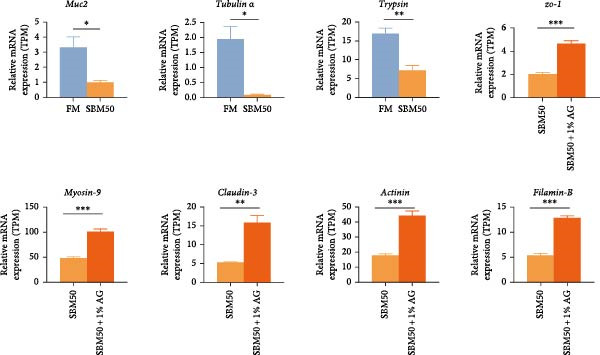
(G)

### 3.7. qPCR Analysis

qPCR validation of transcriptome‐derived DEGs demonstrated that *occludin* and *p50* expression decreased markedly in the SBM50 versus the FM (*p* < 0.05; Figure 7A), whereas *zo-1*, *filamin*, and *desmin* expression were markedly higher in the SBM50 + 1% AG than the SBM50 (*p* < 0.05; Figure 7B). However, the qPCR result of *zo-1* in the SBM50 group was inconsistent with RNA‐seq (Figure 7A).

Figure 7qPCR validation of RNA‐seq identified DEGs. (A) The results of SBM50 versus FM. (B) The results of SBM50 + 1% AG versus SBM50. *zo-1*: *zonula occludens-1*. *p50*: *nuclear factor kappa B subunit 1*.

**Figure:**
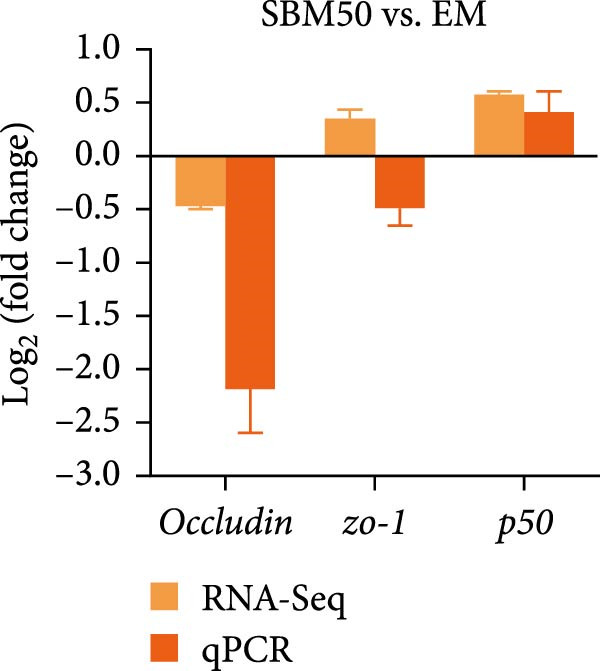
(A)

**Figure:**
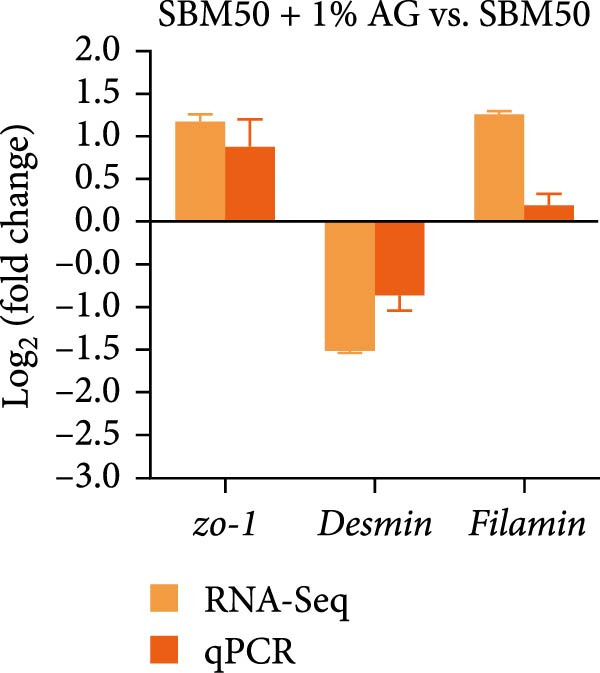
(B)

## 4. Discussion

### 4.1. Effects of Dietary Glutamine on Growth Performance in Juvenile Largemouth Bass Fed High SBM Diets

Growth performance indicated that the SBM50 exhibited reduced FBW, WGR, and SGR. Conversely, the SBM50 + 1% AG markedly increased FBW, WGR, and SGR more than the SBM50. This aligns with findings in hybrid sturgeon (*Acipenser baerii* ♀ × *A. schrenckii* ♂) [23] and zebrafish [24], where 1% AG enhanced growth performance in high SBM diets. Furthermore, studies on juvenile common carp (*Cyprinus carpio*) [25] similarly demonstrated optimal growth performance with 1% AG supplementation. Morphometric analysis revealed that glutamine supplementation enhanced both condition factor (CF) and intestinal length in largemouth bass. This aligns with previous findings in hybrid sturgeon (*Acipenser baerii* ♀ × *A. schrenckii* ♂) [23] and Jian carp (*Cyprinus carpio* var. Jian) [26], suggesting glutamine may promote nutrient absorption and utilization, thereby improving somatic indices. Collectively, these demonstrate that Gln supplementation enhances growth performance in largemouth bass under high SBM diets.

### 4.2. Effects of Glutamine on Serum‐Free Amino Acid Metabolic Profiles in Juvenile Largemouth Bass Fed High SBM Diets

Results demonstrated that 1% AG increased the total essential amino acids. Glycine concentration was markedly higher in the SBM50 + 1% AG than in SBM50. As a non‐essential amino acid, glycine enhances intestinal health and stress resistance in animals, fulfilling broad and critical physiological functions [27]. Studies on rainbow trout [28] revealed that dietary glycine supplementation improves nutrient digestibility, increases bile acid cycling frequency, and may serve as an energy substrate. Gln enhances nutrient utilization efficiency by promoting the deposition of proteins, lipids, and minerals in fish, thereby improving feed efficiency [29, 30]. Consistent findings were observed in juvenile grass carp (*Ctenopharyngodon idella*) [31] and Senegalese sole (*Solea senegalensis*) larvae [32]; Gln supplementation enhanced intestinal protein metabolism, accelerating larval growth. In the present study, high SBM inclusion induced intestinal damage in largemouth bass, and the upregulated glycine concentration may represent a compensatory response to SBMIE. Collectively, these findings indicate that Gln enhances nutrient absorption and utilization efficiency in largemouth bass.

### 4.3. Effects of Dietary Glutamine on Intestinal Histomorphology in Juvenile Largemouth Bass Fed High SBM Diets

H&E and PAS analyses revealed that the SBM50 exhibited significantly reduced MLT and PH versus the FM. In contrast, the SBM50 + 1% AG markedly increased PH, consistent with previous findings in turbot [33] and hybrid grouper [12]. These demonstrate that Gln improves the intestinal morphological damage of largemouth bass induced by high‐dose SBM. The intestine is the major organ for digestion and absorption in fish [34]. Maintaining its structural integrity is crucial for growth and development. The increased PH following AG supplementation indicates expanded absorptive surface area, thereby enhancing nutrient assimilation efficiency and ultimately improving growth performance. Goblet cells, which secrete mucins to form the intestinal mucosal barrier [35], exhibited a decreased number following SBM supplementation, suggesting a compromised intestinal barrier function. TEM further demonstrated severe ultrastructural pathologies in the SBM50 group. These pathological alterations were substantially mitigated in the SBM50 + 1% AG group. The nucleus, as the cellular control center, maintains structural and functional integrity, and nuclear abnormalities compromise cellular function [36]. The endoplasmic reticulum is central to protein folding and transport, cellular homeostasis, and structural abnormalities of the endoplasmic reticulum can lead to apoptosis [37]. Mitochondria, as energy factories, abnormalities in their structure and function can induce the occurrence of diseases [38]. Collectively, Gln supplementation preserves intestinal cytoarchitecture and maintains physiological homeostasis in largemouth bass subjected to high SBM diets.

### 4.4. Effects of Dietary Glutamine on Intestinal Immunity in Juvenile Largemouth Bass Fed High SBM Diets

The complement system constitutes a critical immune defense mechanism, with complement components C3 and C4 playing pivotal roles in fish innate immunity [39]. Although no statistically significant differences in C3 or C4 levels were observed among groups, C4 concentrations were numerically higher in the SBM50 + 1% AG group compared to SBM50. Notably, supplementation with 1% Ala‐Gln significantly reduced caspase3 levels. This aligns with findings in snakehead (*Channa argus*) [15], where Gln supplementation alleviates apoptosis by reducing the gene expression of *caspase3*. AMPs, endogenous peptides produced by the host, serve essential functions in innate immunity [40]. While AMPs expression did not differ significantly across groups, AMPs levels were numerically elevated in the SBM50 + 1% AG group relative to SBM50. Collectively, these results indicate that dietary Gln enhances immune competence in largemouth bass and exhibits potential for apoptosis prevention.

### 4.5. Effects of Dietary Glutamine on Intestinal Microflora in Juvenile Largemouth Bass Fed High SBM Diets

The SBM50 + 1% AG group exhibited markedly higher microbial diversity and richness compared to both the FM and SBM50 groups. This effect is likely mediated by Gln’s role as a key energy source, which maintains barrier integrity and provides a stable colonization environment for commensal bacteria [41]. At the phylum level, the SBM50 group showed abnormal enrichment of Proteobacteria. AG supplementation significantly reduced Proteobacteria abundance while increasing Bacteroidota abundance. This aligns with LEfSe analysis indicating dominance of Bacteroidota and *Prevotella* in the SBM50 + 1% AG group. Elevated Proteobacteria abundance is a recognized biomarker of dysbiosis and negatively correlates with host health status [42]. Conversely, Bacteroidota enhances carbohydrate and protein metabolism, potentially mitigating the adverse effects of high SBM diets through improved immune function and metabolic regulation [43]. At the genus level, significant enrichment of *Bacteroides*, *Treponema*, and *Prevotellaceae_UCG-001* in the SBM50 + 1% AG group carries physiological importance. *Bacteroides* ferments complex polysaccharides, yielding short‐chain fatty acids, strengthening intestinal barrier function [44]. *Prevotellaceae_UCG-001* is associated with anti‐inflammatory effects and dietary fiber metabolism [45]. Venn diagram analysis demonstrated significantly higher unique OTUs in the SBM50 + 1% AG group, suggesting AG not only modulates core microbiota but may also suppress opportunistic pathogens through nutritional competition or niche occupation. LEfSe analysis further confirmed Enterobacteriaceae dominance in the SBM50 group, consistent with soybean‐meal‐induced dysbiosis. AG supplementation restructured the microbial community by enriching Bacteroidota and *Prevotella*. Collectively, dietary supplementation with 1% AG mitigates the adverse effects of high SBM diets on the intestinal microbiota of juvenile largemouth bass by enhancing microbial diversity and richness, suppressing potential pathogens, and improving intestinal health.

### 4.6. Effects of Dietary Glutamine on Intestinal Transcriptome in Juvenile Largemouth Bass Fed High SBM Diets

Transcriptomic analysis revealed that dietary AG supplementation significantly reversed SBM‐induced gene expression dysregulation and ameliorated host health by activating intestinal barrier‐related pathways. Among intestinal barrier genes, significant downregulation of *tubulin α*, *trypsin-2*, and *muc2* in the SBM50 group indicated mechanical barrier impairment via cytoskeletal disruption and diminished MLT [46, 47]. Conversely, the SBM50 + 1% AG group exhibited upregulation of *zo-1*, *claudin-3*, and *filamin-B*, demonstrating AG‐mediated restoration of epithelial barrier integrity through enhanced stability of intercellular junctional complexes. Furthermore, elevated expression of *lamin-A* and *integrin α* suggests AG may maintain epithelial polarity and repair capacity via extracellular matrix‐cytoskeleton signaling [48]. These findings align with turbot [13] studies where AG increased tight junction gene expression under soybean‐meal stress and grass carp [49] research confirming AG‐induced upregulation of intestinal barrier factors. Pathway enrichment analysis showed activated *Vibrio cholerae* infection pathways in the FM and SBM50 groups, potentially linked to *muc2* suppression and heightened pathogen translocation risk. Concurrent activation of arachidonic acid metabolism pathways may exacerbate inflammatory responses, corroborating the detrimental effects of high SBM on intestinal homeostasis. In contrast, the significantly enriched focal adhesion, cell adhesion molecules, and adherens junction pathways in the SBM50 + 1% AG group revealed the mechanism of action of AG at the systemic level. These pathways establish a multi‐tiered barrier repair network by integrating extracellular matrix signals (*integrin α*), regulating intercellular junction proteins (*zo-1*, *claudin-3*), and coordinating cytoskeletal remodeling (*filamin-B*, *actinin*).

## 5. Conclusion

Dietary inclusion of high‐level SBM induced intestinal dysfunction and growth retardation in juvenile largemouth bass. Supplementation with 1% AG ameliorated SBMIE. The underlying mechanisms involve AG‐mediated remodeling of the intestinal microbiota, which notably enriches potentially beneficial bacterial taxa. At the molecular level, AG enhances tight junction‐related gene expression, thereby improving intestinal barrier integrity. Collectively, AG enhances immune competence, promotes amino acid metabolism, and alleviates intestinal inflammation induced by high‐SBM diets. However, the deeper mechanism through which AG mitigates soybean meal‐induced enteritis in largemouth bass remains to be further elucidated.

## Ethics Statement

The study was approved by the Institutional Animal Care and Use Committee of Southwest University (approval number SWU_LAC2024020050).

## Conflicts of Interest

The experimental fish were purchased from Chongqing Sanxia Ecological Fisheries Co., Ltd. but it had no involvement in any other aspect of the study.

## Author Contributions

**Xinpeng Wang**: data curation, formal analysis, visualization, validation, writing – original draft. **Rongyan Yue**: project administration, data curation, software, formal analysis. **Jun Wen**: resources, investigation. **Haiqing Wu**: resources, investigation. **Xinghua Zhou**: supervision, methodology. **Yongjun Chen**: supervision, methodology. **Li Luo**: supervision, methodology. **Shimei Lin**: supervision, visualization. **Qinghui Ai:** supervision, software. **Yuanfa He:** writing – review & editing, methodology, project administration, funding acquisition. Co‐first author Rongyan Yue contributed equally to the first author.

## Funding

This study was financially supported by the National Natural Science Foundation of China (32202951) and the Fundamental Research Funds for the Central Universities (SWU‐KQ22069).

## Data Availability

Data will be made available on request.
